# Valve-Preserving Repair of Very Late Type A Aortic Dissection Following Self-Expanding TAVI in an Octogenarian Patient: Case Report and Focused Narrative Review of the Literature

**DOI:** 10.3390/jcdd13090410

**Published:** 2026-08-24

**Authors:** Lorenzo Giovannico, Giuseppe Fischetti, Domenico Parigino, Luca Savino, Claudia Leo, Giuseppe Cristiano, Giuseppe Scrascia, Massimiliano Carrozzini, Massimo Padalino, Tomaso Bottio

**Affiliations:** Cardiac Surgery Unit, Department of Precision and Regenerative Medicine and Ionian Area (DiMePRe-J), University of Bari Aldo Moro, Piazza Giulio Cesare 11, 70124 Bari, Italy; giuseppe.fischetti@policlinico.ba.it (G.F.); domenico.parigino@policlinico.ba.it (D.P.); luca.savino@policlinico.ba.it (L.S.); c.leo@studenti.uniba.it (C.L.); giuseppecristiano88@gmail.com (G.C.); giuseppe.scrascia@policlinico.ba.it (G.S.); massimiliano.carrozzini@policlinico.ba.it (M.C.); massimo.padalino@uniba.it (M.P.); tomaso.bottio@uniba.it (T.B.)

**Keywords:** transcatheter aortic valve implantation (TAVI), acute type A aortic dissection, late TAVI complications, valve-preserving aortic surgery, self-expanding transcatheter heart valve

## Abstract

**Introduction:** Acute type A aortic dissection (ATAAD) is a rare but potentially catastrophic complication following transcatheter aortic valve implantation (TAVI). Most reported cases occur during or shortly after the procedure and are attributed to procedural aortic injury. Very late presentations occurring years after successful TAVI are exceptionally uncommon, and evidence regarding their optimal management remains limited. **Case Presentation:** An 86-year-old man presented with acute chest pain four years after transfemoral implantation of a self-expanding Evolut R 29-mm transcatheter heart valve. Transthoracic echocardiography revealed pericardial effusion with signs of impending cardiac tamponade. Computed tomography angiography confirmed Stanford type A acute aortic dissection involving the ascending aorta. Emergency surgical repair was performed through replacement of the ascending aorta and hemiarch using a vascular graft. The previously implanted transcatheter valve was preserved because it remained structurally intact and functionally normal. The postoperative course was uneventful, and the patient was discharged on postoperative day 9 with preserved prosthetic valve function (mean gradient 11 mmHg, peak velocity of 2.1 m/s, EOA 1.8 cm^2^, EF 50%, TAPSE 18 mm and no evidence of paravalvular or intraprosthetic regurgitation). **Discussion:** To better contextualize this rare presentation, a focused review of the literature on delayed and late ATAAD after TAVI was performed. Only a limited number of cases were identified, highlighting the exceptional rarity of this complication. Reported management strategies included conservative treatment, endovascular interventions, and open surgical repair, with considerable heterogeneity in outcomes. Compared with previously published reports, the present case is notable for the exceptionally long interval between TAVI and dissection onset and for the successful valve-preserving surgical repair. These findings suggest that emergency surgery with preservation of a functioning transcatheter valve may be a feasible option in carefully selected patients. **Conclusions:** Very late ATAAD after TAVI is an exceptionally rare but life-threatening condition. This case demonstrates that valve-preserving surgical repair can be successfully performed even in selected octogenarian patients. As the population of long-term TAVI survivors continues to expand, awareness of late aortic complications, prompt diagnosis, and referral to specialized aortic centers remain essential for achieving favorable outcomes.

## 1. Introduction

Transcatheter aortic valve implantation (TAVI) has become an established treatment for severe aortic stenosis across a wide range of surgical risk profiles [1]. As long-term survival after TAVI improves, late complications are increasingly recognized. Acute type A aortic dissection (ATAAD) is a rare but potentially fatal complication, most commonly occurring during or shortly after the procedure [2]. Very late ATAAD developing years after successful TAVI remains exceptionally uncommon, and evidence regarding its optimal management is limited. We report the case of an 86-year-old patient who developed ATAAD four years after implantation of a self-expanding Evolut R prosthesis and underwent successful emergency surgical repair with preservation of the transcatheter valve. To place this rare presentation in context, we also review the currently available literature on delayed and late ATAAD after TAVI.

## 2. Case Presentation

**Figure 1:**
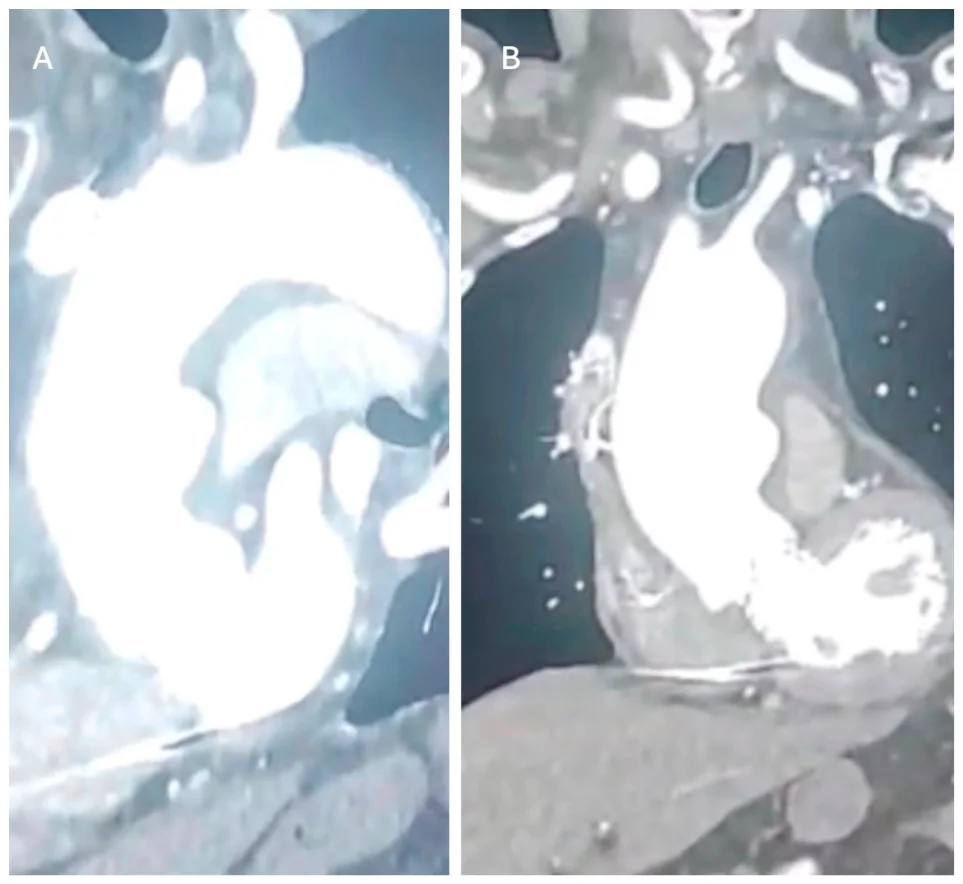
(**A**,**B**). Computed tomography angiography revealed Stanford type A acute aortic dissection involving the ascending aorta.

**Figure 2:**
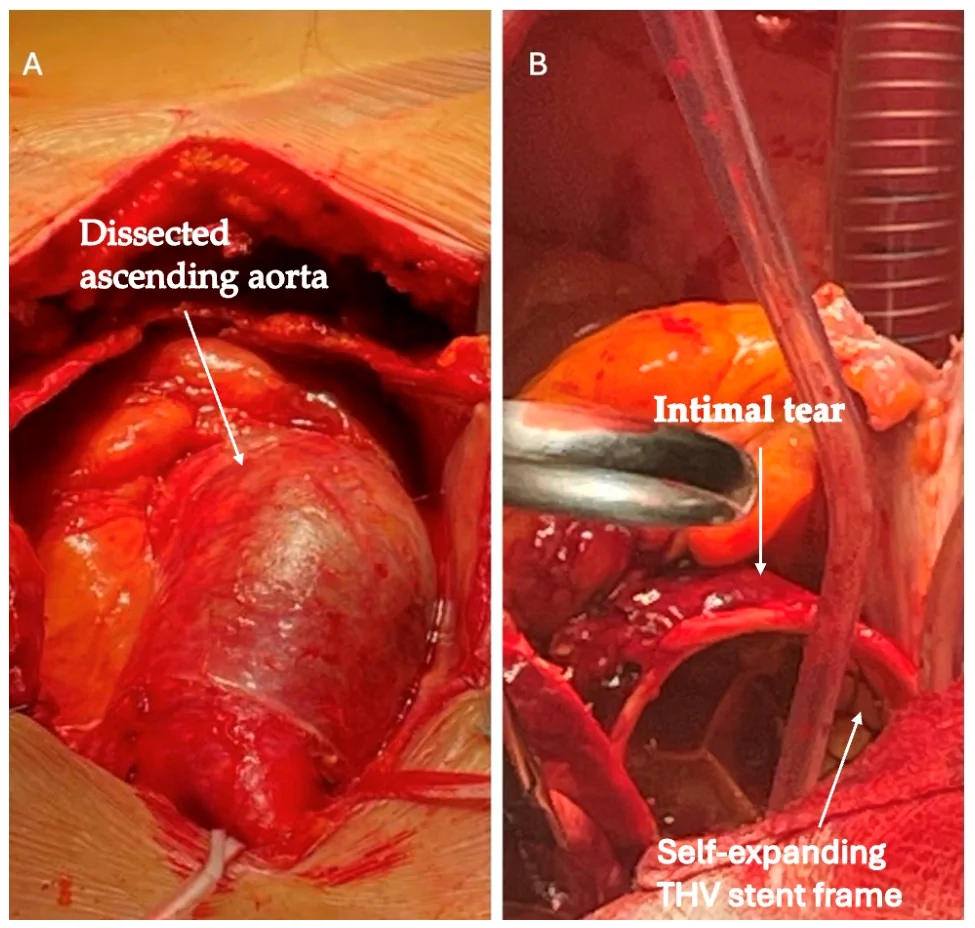
(**A**). The ascending aorta shows signs of aortic dissection and an intramural hematoma. (**B**). An intimal tear located at the level of the previously implanted self-expanding Evolut R stent frame.

**Figure 3:**
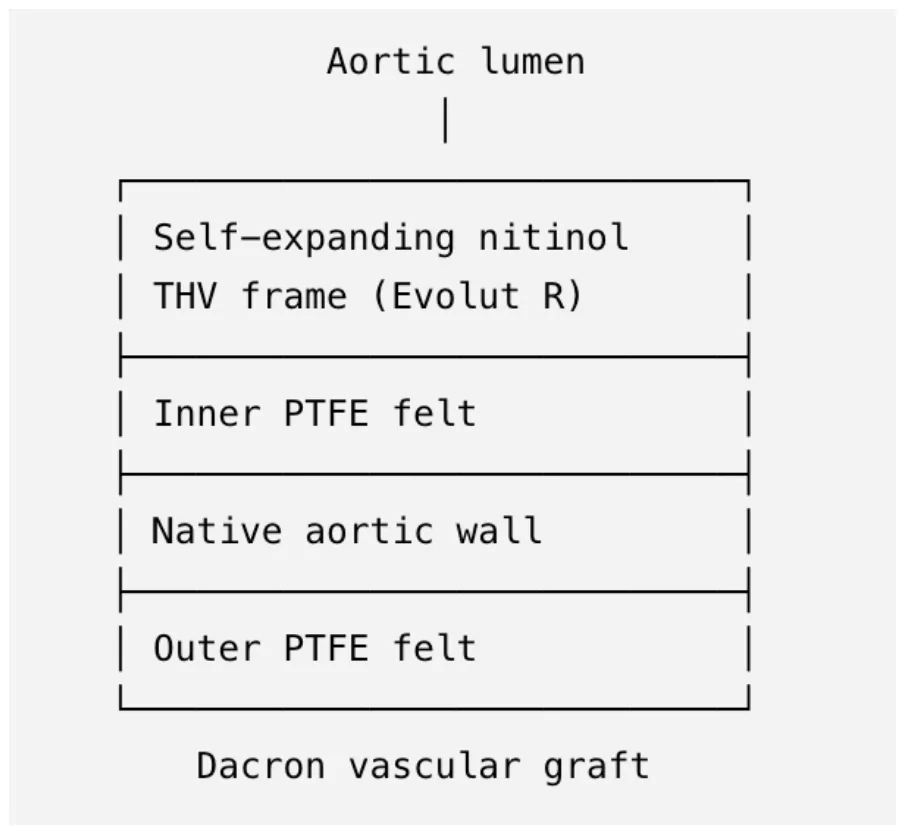
Schematic illustration of the proximal aortic reconstruction. The inner polytetrafluoroethylene (PTFE) felt reinforcement is positioned between the self-expanding transcatheter heart valve (THV) stent frame and the native aortic wall, while the outer PTFE felt reinforcement is placed externally around the aortic wall. The proximal anastomosis is completed with a 30-mm Dacron vascular graft, creating a sandwich reinforcement that provides secure hemostasis while preserving the previously implanted transcatheter valve.

An 86-year-old male with a history of hypertension, dyslipidemia, polycythemia vera receiving ruxolitinib therapy, pulmonary emphysema, hepatic steatosis, and severe calcific aortic stenosis underwent transfemoral TAVI in December 2021 with implantation of a self-expanding Evolut R 29-mm bioprosthesis. At the time of presentation with acute type A aortic dissection, he was receiving dual antiplatelet therapy with aspirin and clopidogrel. Platelet count at presentation was 225 × 10^9^/L. No hematology-specific perioperative intervention was required. There was no known family history of aortic dissection or hereditary aortic disease. Preprocedural multidetector computed tomography demonstrated a tricuspid severely calcified degenerative aortic valve with an annular perimeter of 78 mm (area 480 mm^2^; mean diameter 24.7 mm). The ascending aorta measured 37 mm in diameter, with no evidence of aneurysmal dilatation. Mild calcification extended to the annulus, whereas only minimal calcification was present within the left ventricular outflow tract (LVOT). No significant calcification involved the sinotubular junction or ascending aortic wall. The aortic root anatomy was considered suitable for implantation of a 29-mm self-expanding Medtronic Evolut R prosthesis, resulting in an estimated oversizing of approximately 12%. The aortic angulation was approximately 52°, allowing favorable coaxial alignment during valve deployment. The index TAVI procedure was performed via a transfemoral approach under conscious sedation. Balloon pre-dilatation and post-dilatation were not required. The valve was deployed in a controlled fashion without the need for recapture or repositioning, achieving a final implantation depth of approximately 4 mm below the non-coronary cusp and 5 mm below the left coronary cusp. Final angiography and intra-procedural echocardiography confirmed optimal prosthesis expansion and positioning, with preserved coronary flow, trivial/no paravalvular regurgitation, and no evidence of aortic injury, annular rupture, aortic dissection, or other structural procedural complications. The only peri-procedural complication was the development of complete atrioventricular block, which required implantation of a dual-chamber permanent pacemaker. No other conduction disturbances, vascular complications, coronary obstruction, or procedural adverse events occurred. The patient remained clinically stable during follow-up. A surveillance contrast-enhanced computed tomography scan performed four months before the acute event demonstrated mild interval enlargement of the ascending aorta to 40 mm, with a sinotubular junction diameter of 38 mm, without evidence of intimal flap, intramural hematoma, penetrating aortic ulcer, or other imaging features suggestive of impending aortic dissection. At the most recent echocardiographic evaluation in December 2025, the 29-mm Evolut R valve showed normal hemodynamic performance, with a mean gradient of 7 mmHg, peak velocity of 2.0 m/s, effective orifice area (EOA) of 1.9 cm^2^, TAPSE 19 mm and a left ventricular ejection fraction of 50%. In January 2026, 49 months after TAVI, he presented to a local emergency department with acute-onset chest pain. Echocardiographic evaluation revealed preserved function of the previously implanted TAVI prosthesis (mean gradient 11 mmHg, peak velocity of 2.1 m/s, EOA 1.8 cm^2^, EF 50%, TAPSE 17 mm and no evidence of paravalvular or intraprosthetic regurgitation). A pericardial effusion was present, with early features consistent with impending cardiac tamponade. Computed tomography angiography revealed Stanford type A acute aortic dissection involving the ascending aorta. The ascending aorta measured 50 mm at the level of maximal dilatation, while the sinotubular junction measured 38 mm (Figure 1A,B). The patient was urgently transferred to our tertiary cardiac surgery center for definitive treatment. On admission, the patient had a Glasgow Coma Scale score of 11, with persistent chest pain and spontaneous respiration (SpO2 92%). Despite norepinephrine support (0.05 μg/kg/min), he showed a tendency toward hypotension (BP 85/55 mmHg), prompting emergency surgery. Following median sternotomy, a large hemorrhagic pericardial effusion was evacuated. Cardiopulmonary bypass was established via right axillary artery cannulation and atrio-caval venous drainage. After aortic cross-clamp, Myocardial protection was achieved using St. Thomas’ Hospital No. 2 crystalloid cardioplegia, administered initially after aortic cross-clamping and subsequently repeated every 20–30 min by selective antegrade infusion directly into the coronary ostia in combination with retrograde delivery through the coronary sinus, providing satisfactory myocardial protection throughout the procedure. Inspection of the ascending aorta revealed an intimal tear and aortic rupture measuring approximately 3 cm in length, located adjacent to the upper crown of the previously implanted self-expanding Evolut R stent frame (Figure 2A,B). The supra-aortic vessels were not involved by the dissection and therefore did not require reconstruction. No residual distal aortic dissection was identified on completion of the repair. The lesion extended through the aortic wall and was associated with active bleeding into the pericardial space. The previously implanted TAVI prosthesis was found to be well seated and functioning normally, without signs of structural damage or displacement. Had the transcatheter prosthesis been structurally damaged, dysfunctional, significantly deformed or displaced, affected by infective endocarditis, or had the dissection involved the aortic root requiring root replacement, prosthesis explantation and surgical aortic valve replacement would have been performed. The self-expanding frame did not interfere with aortic cross-clamping, exposure of the aortic root, selective coronary ostial cardioplegia delivery, or proximal suturing. After resection of the injured aortic segment, the proximal and distal aortic stumps were reinforced using a sandwich technique with Polytetrafluoroethylene (PTFE) felt strips (Figure 3).

**Figure 4:**
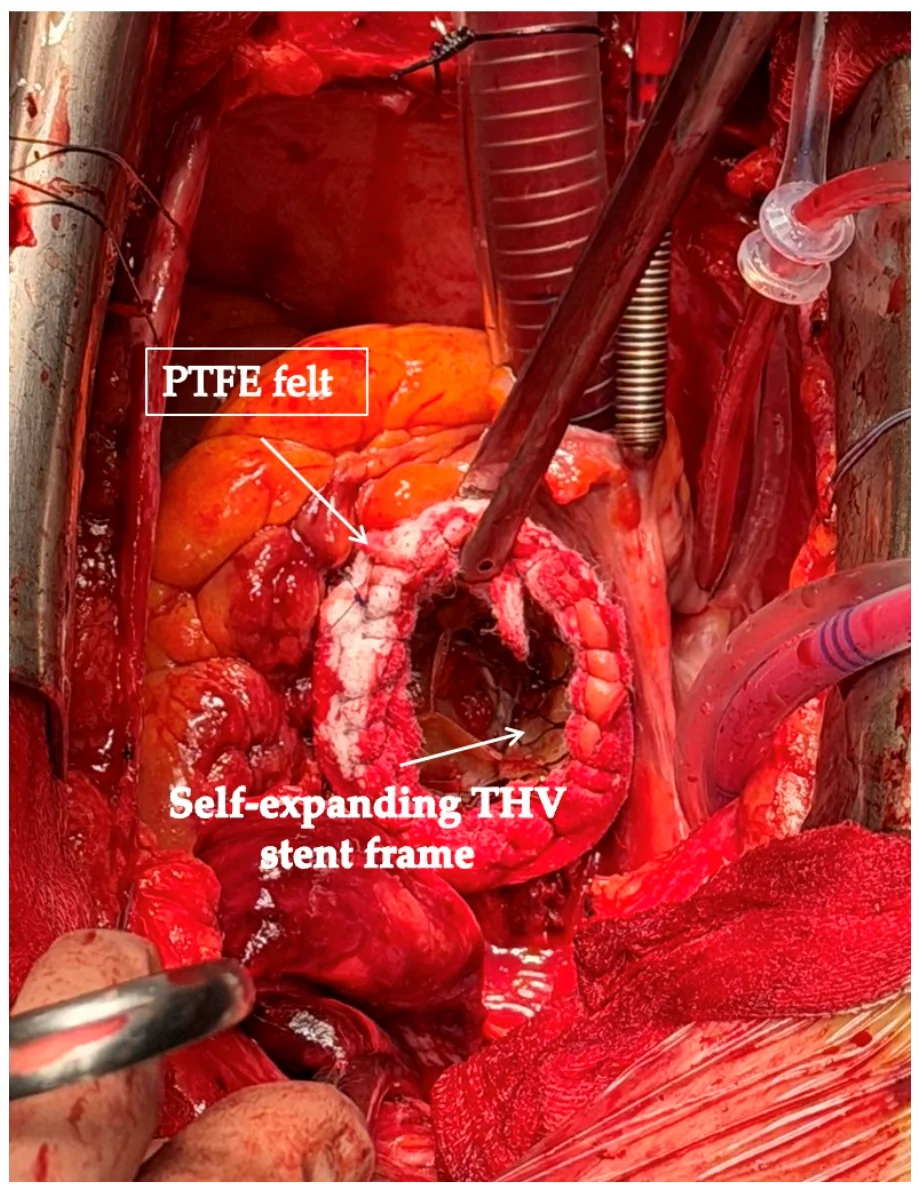
The inner felt strip was positioned between the self-expanding TAVI stent frame and the native aortic wall, while the outer felt strip was placed externally around the aortic wall, providing secure reinforcement of the anastomotic site.

At the proximal anastomosis, the inner felt strip was positioned between the self-expanding TAVI stent frame and the native aortic wall, while the outer felt strip was placed externally around the aortic wall, providing secure reinforcement of the anastomotic site (Figure 4). The ascending aorta was replaced from the distal end of the previously implanted self-expanding transcatheter valve frame to the origin of the brachiocephalic trunk. The distal repair was performed using an open distal anastomosis under circulatory arrest. A limited resection of the inferior aspect (lesser curvature) of the proximal aortic arch was carried out to completely excise the dissected aortic tissue and obtain healthy distal margins, followed by reconstruction with a 30-mm straight tubular Dacron vascular graft. The aortic wall, at histological examination, showed chronic degenerative changes with medial fibrosis, elastic fiber fragmentation, and focal myxoid (proteoglycan-rich) degeneration, consistent with age-related medial degeneration. No inflammatory infiltrates or features suggestive of connective tissue disease were observed. These histopathological findings are consistent with age-related medial degeneration rather than a specific TAVI-related lesion. The lowest temperature reached during cardiopulmonary bypass was 29 °C. Circulatory arrest time was 29 min, total cardiopulmonary bypass time was 177 min, and aortic cross-clamp time was 83 min. The patient required transfusion of three units of packed red blood cells and one apheresis platelet concentrate. The transcatheter aortic valve was carefully evaluated by intraoperative transesophageal echocardiography both before initiation of cardiopulmonary bypass and after completion of the surgical repair. Preoperative assessment demonstrated a well-functioning transcatheter heart valve with normal leaflet mobility, no evidence of structural valve deterioration, prosthetic thrombosis, paravalvular or transvalvular regurgitation, and no echocardiographic signs of infective endocarditis. Given the satisfactory prosthetic valve function and the absence of valve-related pathology, preservation of the previously implanted transcatheter valve was considered the most appropriate surgical strategy. Valve explantation and surgical aortic valve replacement would have substantially increased operative complexity, cardiopulmonary bypass and aortic cross-clamp times, thereby exposing this elderly patient to a significantly higher operative risk. Intraoperative transesophageal echocardiography performed after completion of the repair confirmed preserved prosthetic valve function, normal leaflet motion, the absence of transvalvular or paravalvular regurgitation, and unchanged transvalvular hemodynamic performance.

**Figure 5:**
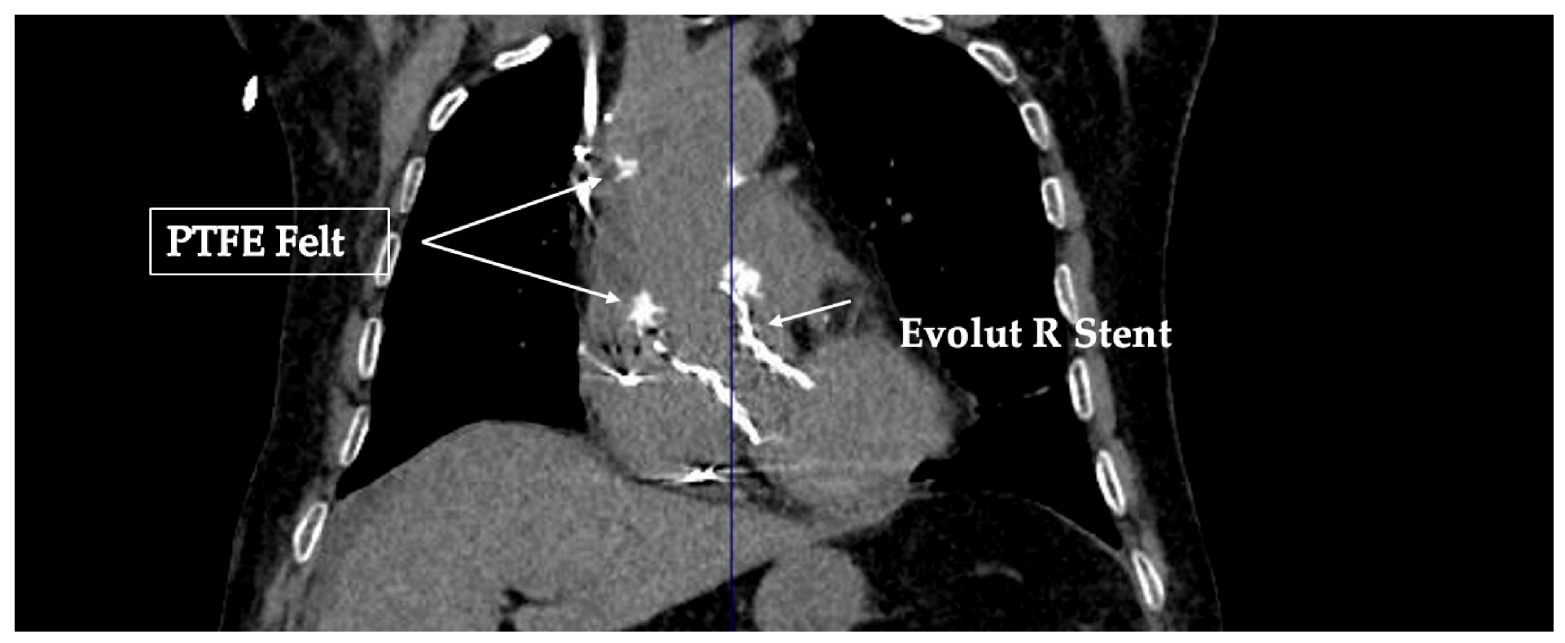
Postoperative CTA showing successful ascending aortic replacement with preservation of the previously implanted self-expanding TAVI prosthesis. Proximally, a Teflon felt strip is interposed between the transcatheter valve stent frame, the native aortic wall, and the vascular graft as part of the sandwich reinforcement technique. Distally, a second Teflon felt strip is positioned between the vascular graft and the native aortic wall, providing additional anastomotic support.

**Figure 6:**
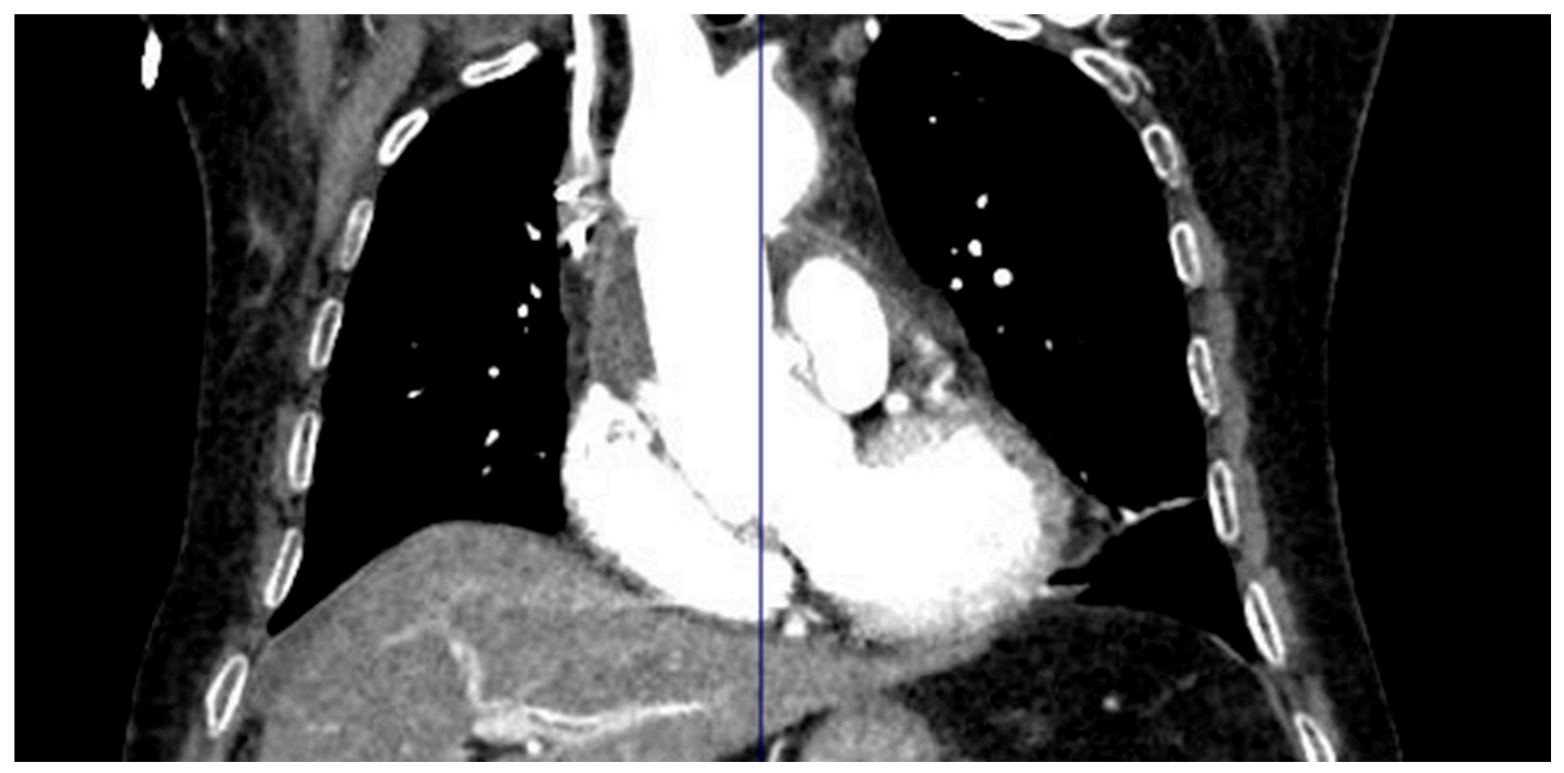
Postoperative contrast-enhanced chest CTA showing successful ascending aortic repair with the vascular graft and previously implanted TAVI prosthesis in situ. No contrast extravasation, residual dissection, anastomotic complications, or pericardial effusion are observed.

**Figure 7:**
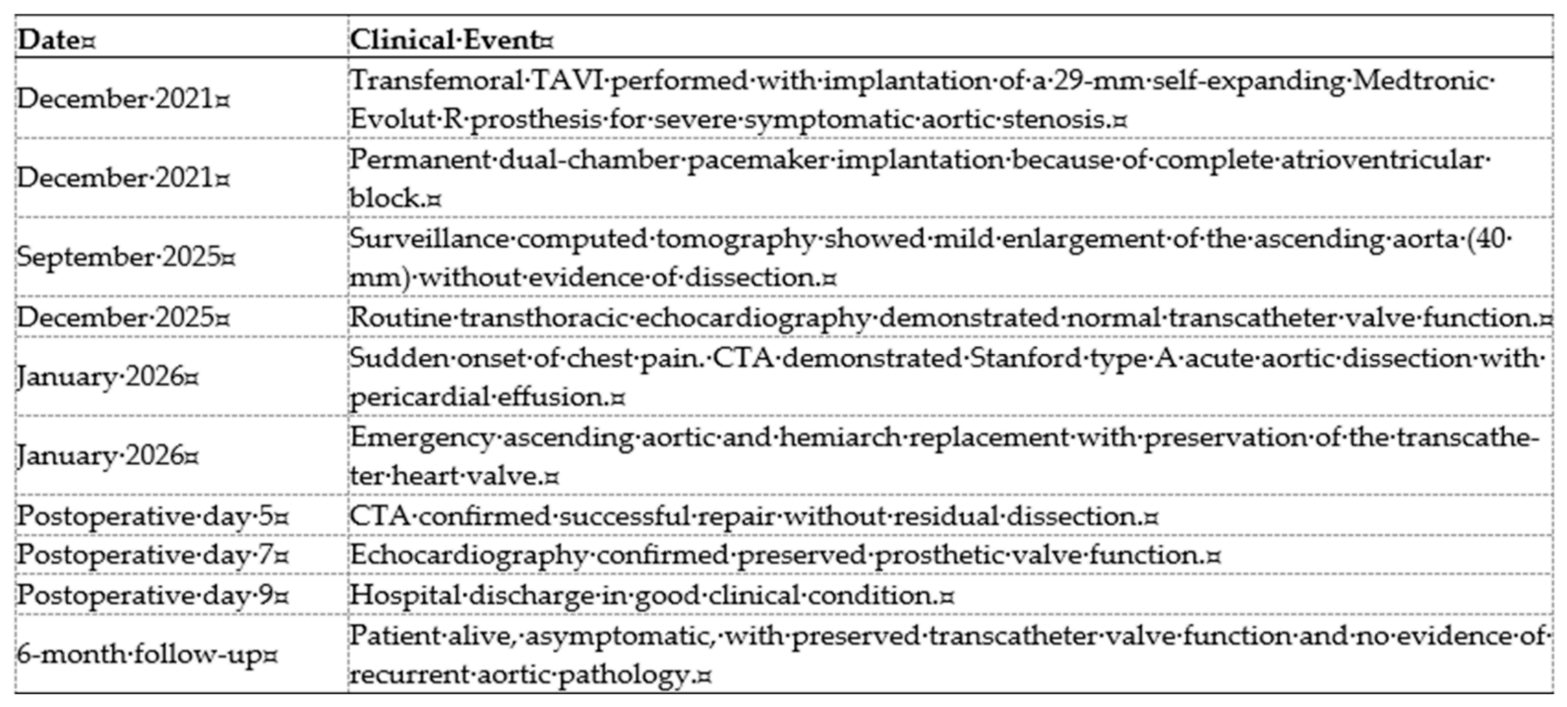
Timeline summarizing the patient’s clinical course from the index TAVI procedure through the diagnosis of very late Stanford type A acute aortic dissection, emergency surgical repair, postoperative recovery, and six-month follow-up.

The postoperative course was uneventful. The patient was successfully extubated 12 h after surgery and subsequently demonstrated progressive clinical improvement. Considering the underlying diagnosis of polycythemia vera, ruxolitinib (Jakavi^®^) therapy was temporarily withheld perioperatively and resumed 12 h after extubation. No additional hematology-specific perioperative interventions were required. Echocardiography confirmed normal function of the previously implanted TAVI prosthesis, with hemodynamic parameters comparable to those observed before surgery. The postoperative course was uneventful, with no major neurological, renal, or respiratory complications. On postoperative day 5, the patient underwent follow-up computed tomography angiography (CTA) of the chest, which confirmed satisfactory repair of the ascending aorta and aortic arch, with no evidence of residual dissection, anastomotic complications, or pericardial effusion (Figure 5 and Figure 6). Pre-discharge transthoracic echocardiography performed on postoperative day 7 demonstrated preserved function of the transcatheter aortic valve prosthesis, with a mean transvalvular gradient of 11 mmHg, peak velocity of 2.1 m/s, and an effective orifice area of 1.8 cm^2^. Left ventricular ejection fraction was 50%, TAPSE was 18 mm, and no paravalvular or intraprosthetic regurgitation was detected. The patient was transferred to the general ward on postoperative day 2, progressively regained functional autonomy, and was discharged on postoperative day 9. (Figure 7) At discharge, medical therapy included aspirin 100 mg once daily, ramipril 5 mg once daily, and amlodipine 5 mg once daily, together with enoxaparin 4000 IU twice daily for 15 days. Strict blood-pressure control was recommended, with a target blood pressure <130/80 mmHg. At six-month follow-up, the patient remained alive, clinically stable, and living independently at home. Follow-up echocardiography confirmed preserved function of the transcatheter aortic valve prosthesis without evidence of structural deterioration or significant regurgitation.

## 3. Focused Narrative Review

To better contextualize the present case, we performed a focused narrative review of the literature using predefined eligibility criteria. PubMed/MEDLINE was searched from database inception to 31 May 2026. The search strategy included combinations of the following terms: (“transcatheter aortic valve implantation” OR “TAVI” OR “transcatheter aortic valve replacement” OR “TAVR”) AND (“acute type A aortic dissection” OR “Stanford type A dissection” OR “aortic dissection”). Additional relevant publications were identified through manual screening of the reference lists of retrieved articles. Two reviewers independently screened the retrieved records for eligibility and independently extracted data from the included studies. Any discrepancies were resolved through discussion and consensus.

**Figure 8:**
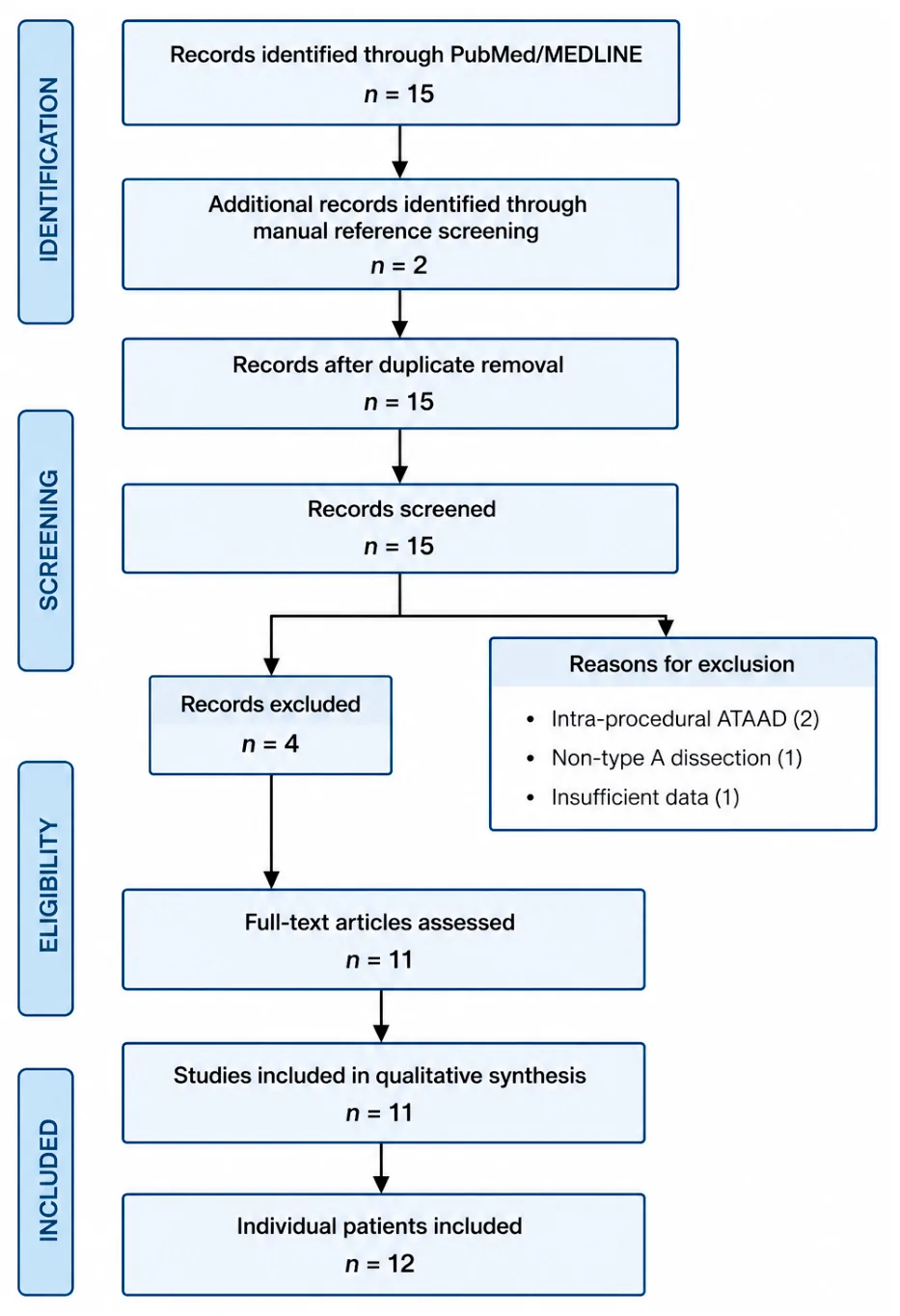
PRISMA flow diagram illustrating the identification, screening, eligibility assessment, and inclusion process of studies reporting delayed or late Stanford type A acute aortic dissection following TAVI.

The search identified 15 records through PubMed/MEDLINE and two additional records through manual screening of reference lists. After removal of duplicates and application of the predefined eligibility criteria, 11 studies describing 12 individual patients with delayed or late Stanford type A acute aortic dissection following TAVI/TAVR were included in the qualitative synthesis. Only English-language publications were included (Figure 8).

We defined delayed ATAAD as acute type A aortic dissection occurring more than 24 h after the index TAVI/TAVR procedure, whereas late ATAAD referred to presentations occurring months or years after valve implantation. Reports describing delayed or late Stanford type A acute aortic dissection following TAVI/TAVR were considered eligible. Cases occurring during the index procedure and reports lacking sufficient clinical information were excluded.

Data extracted from eligible reports included patient demographics, transcatheter heart valve type, interval between TAVI/TAVR and dissection onset, presumed entry tear location, treatment strategy, prosthesis preservation, and clinical outcome.

## 4. Results of the Literature Review

A total of 11 studies describing 12 patients with delayed or late Stanford type A acute aortic dissection following TAVI/TAVR were identified. Most reported cases involved self-expanding transcatheter heart valves, and the ascending aorta represented the predominant location of the primary entry tear. Conservative treatment was associated with uniformly poor outcomes, whereas favorable outcomes were mainly observed after surgical or endovascular intervention. Valve preservation during surgery was feasible in selected patients with preserved prosthetic function. The longest interval between TAVI and ATAAD was observed in the present case (49 months). Detailed patient characteristics and aggregated findings are summarized in Table 1 and Table 2.

**Table 1:** Published Cases of Delayed or Late Stanford Type A Aortic Dissection After TAVI/TAVR [3,4,5,6,7,8,9,10,11,12].

Author	Year	Age/Sex	Valve Type	Time from TAVI	Entry Tear Location	Treatment	THV Preserved	Outcome
Gerber et al. (Case 1) [3]	2010	NR	CoreValve	Several days	Ascending aorta	Conservative	NA	Death
Gerber et al. (Case 2) [3]	2010	NR	CoreValve	Several days	Ascending aorta	Conservative	NA	Death
Al-Attar et al. [5]	2013	NR	CoreValve	8.5 months	Adjacent to valve frame	Conservative	NA	Death
Conzelmann et al. [6]	2023	Elderly male	CoreValve	4 weeks	Ascending aorta (DeBakey I)	Conservative	NA	Death
Losmanova et al. [7]	2018	NR	Self-expanding THV	Delayed	Ascending aorta	Conservative	NA	Death
Pontious et al. [8]	2020	NR	Evolut	12 months	Ascending aorta/Arch	Frozen elephant trunk	NR	Survived
Brinkmann et al. [9]	2021	NR	Evolut R 34 mm	5 months	Proximal ascending aorta	Endovascular closure	Yes	Survived
Fujita et al. [10]	2023	NR	Self-expanding THV	2 days	Ascending aorta	Conservative	NA	Death
Naar et al. [11]	2023	NR	TAVR prosthesis	12 months	Adjacent to prosthesis	Surgical repair	NR	Death
Şahin et al. [12]	2024	NR	ACURATE neo 27 mm	1 week	Ascending aorta	Conservative	NA	Death
Shiwaku et al. [4]	2025	NR	Balloon-expandable THV	14 days	Ascending aorta	Surgical repair with THV explantation	No	Survived
Present case	2026	86/M	Evolut R 29 mm	49 months	At level of self-expanding stent frame	Ascending aorta and hemiarch replacement	Yes	Survived

**Table 2:** Summary of published cases of delayed or late Stanford type A acute aortic dissection following TAVI/TAVR. Delayed ATAAD after TAVI/TAVR remains exceptionally rare and predominantly involves self-expanding transcatheter valves. Most reported entry tears originated in the ascending aorta. Conservative management was uniformly associated with mortality, whereas invasive treatment (surgical or endovascular) achieved favorable outcomes in the reported cases.

Variable	Value
Total reported patients (including present case)	12
Median time from TAVI to ATAAD	10.3 months
Longest reported interval	49 months (present case)
Self-expanding valves	7/8 (87.5%)
Balloon-expandable valves	1/8 (12.5%)
Ascending aorta as presumed entry tear location	12/12 (100%)
Open surgical repair	4/12 (33.3%)
Endovascular treatment	1/12 (8.3%)
Conservative treatment	7/12 (58.3%)
THV preserved during intervention	3/4 (75.0%)
Overall survival	8/12 (66.7%)
Mortality after conservative treatment	7/7 (100%)
Mortality after invasive treatment (surgical/endovascular)	1/5 (20%)

## 5. Discussion

Acute Stanford type A aortic dissection (ATAAD) is a rare but potentially catastrophic complication following transcatheter aortic valve implantation (TAVI). The majority of reported cases occur during the procedure or within the early postoperative period and are generally attributed to direct mechanical injury of the aortic wall caused by guidewires, delivery systems, balloon valvuloplasty, or valve deployment [2]. In contrast, delayed and very late presentations occurring months or years after an initially successful TAVI procedure are exceedingly uncommon and remain poorly characterized in the literature [3].

The present case is remarkable because ATAAD developed approximately four years after implantation of a self-expanding Evolut R prosthesis. To the best of our knowledge, only a limited number of delayed or late ATAAD cases following TAVI have been reported. Although a definitive causal relationship between TAVI and subsequent dissection cannot be established, several pathophysiological mechanisms may explain this association. Chronic radial forces exerted by self-expanding transcatheter valve frames have been hypothesized to induce progressive stress on the adjacent aortic wall, potentially contributing to long-term structural alterations [13,14]. In addition, patient-related factors, including persistent systemic hypertension, age-related degeneration of the aortic media, extensive aortic calcification, and underlying aortopathy, may increase susceptibility to aortic wall injury and dissection. The prolonged interval observed in our patient suggests that progressive aortic wall remodeling and degeneration were likely more relevant than procedural factors alone.

The clinical setting in which TAVI is performed may also influence procedural and early outcomes. Urgent or emergent TAVI is increasingly used in patients presenting with acute decompensated heart failure or hemodynamic instability, representing a substantially different clinical scenario from elective TAVI. A recent meta-analysis by Apostolos et al. showed that urgent TAVI was associated with significantly higher in-hospital and 30-day mortality, as well as increased rates of acute kidney injury and vascular complications, compared with elective procedures. More recently, Ktenopoulos et al., in a systematic review and meta-analysis including 71,909 patients, found no significant difference in the incidence of unplanned conversion to cardiac surgery between urgent and elective TAVI, although urgent procedures remained associated with higher in-hospital, 30-day, and 1-year mortality and an increased risk of acute kidney injury. These findings suggest that the poorer outcomes observed after urgent TAVI may predominantly reflect the greater baseline clinical instability and comorbidity burden of these patients rather than procedural urgency itself. Importantly, the index TAVI in our patient was uncomplicated, without evidence of acute aortic injury; therefore, data derived from urgent or emergent TAVI populations should be considered as relevant contemporary context rather than as evidence of a causal mechanism for the very late aortic dissection observed in the present case [15,16].

These patients are frequently elderly and often present with multiple comorbidities, characteristics that originally led to the selection of a transcatheter rather than a surgical approach for treatment of aortic stenosis. Consequently, the risk–benefit balance of emergency surgery may appear unfavorable. Nevertheless, the natural history of untreated ATAAD remains extremely poor, with mortality increasing dramatically during the first hours after symptom onset [17,18]. Contemporary evidence indicates that carefully selected octogenarians may achieve acceptable perioperative and mid-term outcomes following emergency surgical repair, particularly in the absence of severe frailty, advanced organ dysfunction, or extensive malperfusion syndromes [19]. Our experience further supports the concept that chronological age alone should not be considered an absolute contraindication to surgery when a potentially life-saving intervention is technically feasible.

A particularly noteworthy aspect of the present case was the successful preservation of the previously implanted transcatheter valve prosthesis. Removal of a self-expanding TAVI device during emergency aortic surgery can substantially increase operative complexity, prolong cardiopulmonary bypass and aortic cross-clamp times, and necessitate more extensive procedures such as aortic root replacement and valve reconstruction [4,20]. In our patient, the prosthesis remained structurally intact and functionally competent, allowing ascending aorta and hemiarch replacement without valve explantation. This strategy simplified the operation, minimized surgical trauma, and preserved satisfactory valve function. The favorable postoperative course supports the feasibility of a valve-sparing approach in selected patients when the transcatheter prosthesis is not directly involved in the dissection process and demonstrates normal hemodynamic performance.

To place our findings in context, we performed a focused review of published reports describing delayed (>24 h after the index procedure) or late Stanford type A aortic dissection following TAVI (Table 1). The available evidence confirms the rarity of this complication, with only isolated cases reported worldwide. Most previously described dissections occurred either during the procedure or within the first months after valve implantation, whereas presentations beyond one year remain exceptional. Reported intervals have ranged from several days to a few years after TAVI [3,4,5,6,7,8,9,10,11,12]. The ascending aorta has consistently represented the most common site of the primary intimal tear, supporting the hypothesis of a localized interaction between the transcatheter valve frame and the aortic wall in predisposed patients [13].

The therapeutic strategies reported in the literature have been heterogeneous and include conservative management, endovascular interventions, and open surgical repair (Table 2) [4,8,9,11]. Notably, patients treated conservatively generally experienced poor outcomes, reflecting the aggressive natural history of ATAAD [3,5,6,7,10]. In contrast, favorable outcomes have been reported in selected patients undergoing surgical or endovascular treatment [8,9]. Compared with previously published reports, the present case is distinguished by the exceptionally long interval between TAVI and dissection onset (49 months) and by the successful preservation of a normally functioning self-expanding transcatheter valve during emergency open repair. These findings contribute additional evidence supporting individualized surgical decision-making and suggest that preservation of the transcatheter prosthesis may be a viable option when anatomical and functional conditions are favorable.

As the population of TAVI recipients continues to expand and long-term survival improves, clinicians will increasingly encounter complications that were previously considered exceptional [21,22]. The progressive extension of TAVI indications toward younger and lower-risk patients further underscores the importance of understanding the long-term interaction between transcatheter valve prostheses and the native aorta [1,23,24]. Although current evidence remains limited to isolated reports and small case series, awareness of delayed aortic complications is essential for timely diagnosis and appropriate management.

Several limitations should be acknowledged. First, as a single case report, no definitive causal relationship between TAVI and subsequent aortic dissection can be established. Second, the absence of serial imaging specifically assessing progressive aortic wall changes before the acute event limits mechanistic interpretation. Third, conclusions derived from the literature review are constrained by the small number of reported cases and the inherent publication bias associated with rare complications. Nevertheless, the rarity of this condition makes individual observations valuable, particularly when combined with a comprehensive review of previously published experiences.

## 6. Conclusions

Very late Stanford type A acute aortic dissection following transcatheter aortic valve implantation is an exceptionally rare but life-threatening complication. This case demonstrates that emergency ascending aortic and hemiarch replacement with preservation of a well-functioning self-expanding transcatheter heart valve can be successfully performed in carefully selected patients, avoiding the additional complexity associated with valve explantation.

Although the intimal tear was located adjacent to the transcatheter valve frame, this observation should be interpreted as an anatomical association rather than evidence of a causal relationship. Age-related degeneration of the aortic wall and other patient-specific factors likely contributed to the development of the dissection.

Our focused review confirms the rarity of delayed and very late ATAAD after TAVI and highlights the marked heterogeneity of the available evidence. As the population of long-term TAVI survivors continues to grow, clinicians should remain aware of this uncommon complication. Further studies are needed to better define the underlying mechanisms, identify patients at increased risk, and determine whether selected patients may benefit from tailored long-term imaging surveillance.

## Data Availability

The data presented in this study are available on request from the corresponding author.

## Abbreviations

The following abbreviations are used in this manuscript:

ATAAD	Acute Type A Aortic Dissection
CPB	Cardiopulmonary Bypass
CTA	Computed Tomography Angiography
EF	Ejection Fraction
EOA	Effective Orifice Area
IRB	Institutional Review Board
LVEF	Left Ventricular Ejection Fraction
NR	Not Reported
TAPSE	Tricuspid Annular Plane Systolic Excursion
TAVI	Transcatheter Aortic Valve Implantation
TAVR	Transcatheter Aortic Valve Replacement
THV	Transcatheter Heart Valve
